# Ear melanoma: a four-case series^☆^^☆☆^

**DOI:** 10.1016/j.abd.2020.08.003

**Published:** 2020-11-18

**Authors:** Lara Martins Fiorio, Lucia Martins Diniz, Karla Spelta, Bruna Anjos Badaró

**Affiliations:** Hospital Universitário Cassiano Antônio de Moraes, Universidade Federal do Espírito Santo, Vitória, ES, Brazil

**Keywords:** Ear, Melanoma, Prognosis

## Abstract

External ear melanoma is rare, and early diagnosis and treatment are paramount for the patient's survival. Four clinical cases are reported, emphasizing the importance of the routine clinical examination of the ears in the dermatological consultation. The study included male and female patients, aged 60 to 81 years old, with melanocytic lesions in the outer ear, evaluated with detailed physical and dermoscopic examination, leading to the identification of lesions suggestive of melanoma. The cases were treated surgically with excision of the lesion, and the diagnoses were confirmed by histopathological study. The therapeutic approach was instituted early as most cases were diagnosed at an early stage, which directly impacted global survival.

## Introduction

External ear melanoma is rare, corresponding to 1%–4% of cutaneous melanomas, among an incidence of 76,380 cases in 2016 in the United States. It affects individuals preferably in the sixth decade of life, being predominantly observed in white men, in the auricular helix, and of the extensive superficial subtype.^1^, ^2^ Early diagnosis directly impacts survival. There is no consensus on treatment due to the lack of specific protocols; however, there is a tendency to adopt a surgical approach with reduced resection margins.^1^

## Case report

### Case 1

**Figure 1:**
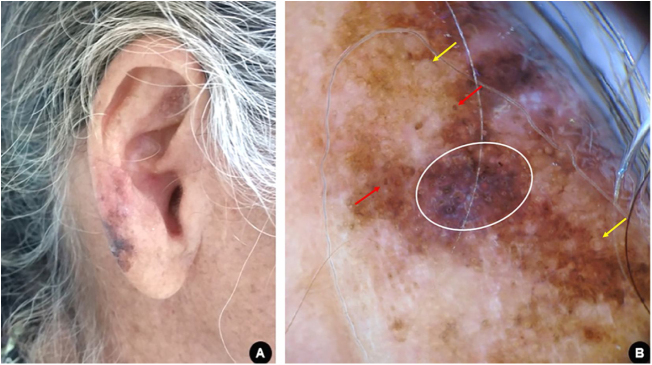
(A), Asymmetric, multicolored, and irregularly contoured pigmented area on the right ear lobe. (B), Dermoscopy suggestive of melanoma, lentigo maligna subtype: homogeneous bluish-gray area with rhomboidal structures (white circle); pigmentation of follicular openings (red arrows); circle within a circle (yellow arrows).

A 60-year-old white woman, farmer, with xeroderma pigmentosum, reported skin cancer since the second decade of life, for which she had previously undergone multiple surgical and non-surgical treatments. The patient underwent clinical follow-up at regular appointments, but was lost to follow-up and resumed after two years. Upon dermatological examination, a brownish macule with irregular color and dermoscopy suggestive of melanoma was observed on the right ear lobe (Fig. 1). The lesion was completely excised with a minimum safety margin, and histopathological examination confirmed the diagnosis of melanoma of the lentigo maligna subtype, with a Breslow thickness of 1 mm. Ulceration, mitosis, and vascular invasion were not observed. Three-dimensional margins were enlarged according to the Breslow thickness. The tumor was classified as stage IA and the patient is currently being followed-up.

### Case 2

**Figure 2:**
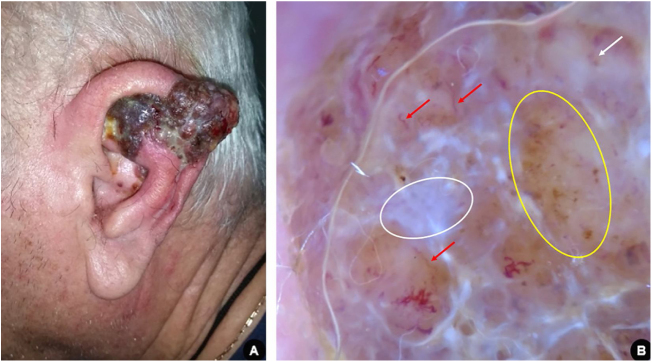
(A), Erythemato-violaceous exophytic tumor affecting the left ear helix with the presence of confluent satellite papules. (B), Dermoscopy suggestive of nodular melanoma: bluish-white veil (white circle); atypical vascular pattern, with irregular linear vessels (red arrows) and milky red globules (white arrow); irregular dots and globules of asymmetric distribution (yellow circle).

Male, 66 years old, white, administrative manager, with medical history of hypertension, dyslipidemia, diabetes, and severe coronary artery disease, sought medical attention due to the onset of a friable tumor on the left ear helix three months prior. The clinical and dermoscopic examination of the lesion was suggestive of nodular melanoma (Fig. 2). The patient was promptly submitted to partial auriculectomy with total excision of the lesion and preservation of the perichondrium and cartilage not affected by the tumor. The histopathological study confirmed invasive nodular melanoma, Breslow thickness of 11 mm, presence of ulceration, and mitotic index of 4 / mm^2^. Tomographic examination revealed an infiltrative lesion located in the subcutaneous area close to the left ear, probably corresponding to the remaining lesion, in addition to regional lymph node involvement. There was no evidence of distant metastatic disease.

Due to the patient's high cardiovascular risk, surgical treatment of the primary tumor with enlargement of margins and sentinel lymph node search was considered unfeasible. Adjuvant radiotherapy was indicated for local control. The tumor was classified as stage IIIC and the patient has been followed-up for ten months, with no signs of disease progression.

### Case 3

**Figure 3:**
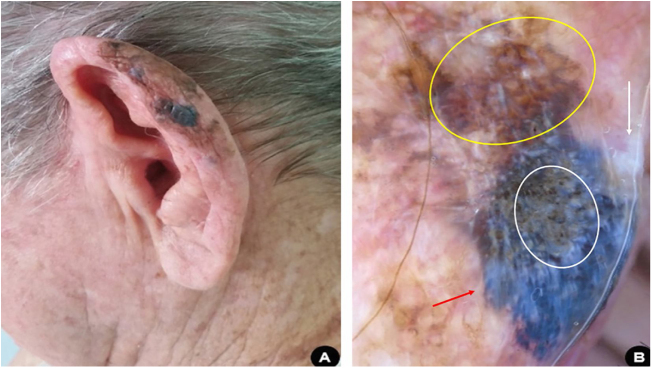
(A), Pigmented, multicolored, asymmetric lesion, with slightly elevated areas on the helix of the left ear. (B), Dermoscopy suggestive of superficial spreading melanoma: blue-white veil (red arrow); irregular dots and globules (white circle); area with rhomboidal structures and asymmetric pigmentation of follicular ostia (yellow circle); white depigmentation area (white arrow).

A 81-year-old white man, driver, noticed the appearance of an asymmetrical pigmented lesion, with irregular borders, on the helix of his left ear, which had evolved for a year and six months asymptomatically. Upon clinical and dermoscopic examination, he presented findings suggestive of melanoma (Fig. 3). An excisional biopsy of the lesion was performed with minimal safety margins, preserving the perichondrium not affected by the tumor. Histopathology confirmed that it was a melanoma, superficial spreading subtype, with a Breslow thickness of 0.8 mm. Ulceration, mitosis, and vascular invasion were not observed. Subsequently, margin enlargement surgery was performed according to the Breslow thickness. The patient was diagnosed at an early stage (IA) and is undergoing clinical follow-up.

### Case 4

**Figure 4:**
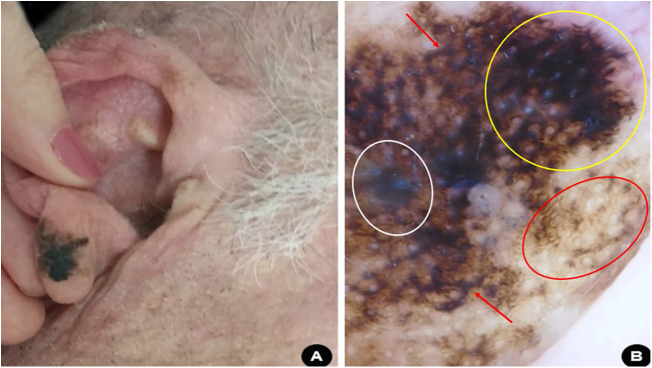
(A), Asymmetric, blackened macula on the right ear lobe. (B), Dermoscopy suggestive of superficial spreading melanoma: bluish-white veil (white circle), homogeneous area with obliterated hair follicles and irregular streaks (yellow circle); rhomboidal structures (red arrow); circle within circle (red circle).

A 69-year-old white man, farmer, underwent regular annual dermatological follow-up after invasive squamous cell carcinoma on his left forearm was excised six years ago. In one of the evaluation consultations, a clinical and dermoscopic alteration was detected: an asymmetric pigmented area on the lobe of the right ear, with findings indicating melanoma (Fig. 4). An excisional biopsy was performed with safety margins and the histopathology assessment confirmed the diagnosis of superficial spreading melanoma, with Breslow thickness of 0.2 mm, and presence of 1 mitoses/mm^2^. Ulceration and vascular invasion were not observed. Then, surgery to expand the safety margins by 1 cm was performed. The tumor was classified as early stage (AI) and the patient is being followed-up.

## Discussion

External ear melanoma (EEM) represents 1% of all primary cutaneous melanomas. The mean age at EEM diagnosis is around the sixth decade of life, and there is a significant predominance in males (86.5%) and fair-skinned individuals.^2^ It is speculated that long hair covering the outer ear could confer a protective effect, justifying the reduced incidence of EEM in women. In addition, men tend to have professional activities more exposed to solar radiation, leading to a higher risk.^2^ The present case series demonstrates findings similar to those in the literature, as the affected woman had xeroderma pigmentosum and was a farmer.

EEM predominantly affects the regions of the helix and ear lobes; the most common subtypes are the superficial spreading (40.1%), followed by the lentigo maligna (33.7%) and nodular (16.4%).^2^, ^3^ Superficial spreading melanoma affects individuals aged 30–40 years and clinically presents as a slightly raised, multicolored area. Lentigo maligna has a peak incidence between the sixth and seventh decades of life, presenting as an irregularly colored patch and affecting areas with extensive actinic damage. Nodular melanoma usually occurs in the fifth decade, as a nodular lesion or elevated plaque, and has an unfavorable prognosis.^4^

Dermoscopy assists the diagnosis and some findings can predict the severity of the lesion, as observed in Case 2, in which the atypical vascular pattern was correlated with the deeper invasion of the tumor in the histopathologic examination.^5^ The diagnosis of most EEMs is usually done in the early stage, with Breslow thickness less than 2 mm in 75% of patients.^1^ Advanced age, greater tumor thickness, presence of ulceration, lymph node involvement, and distant metastasis are the main prognostic predictors, each acting independently on mortality, whereas anatomical location has no impact on tumor recurrence and survival outcomes.^1^, ^2^

The treatment of EEM does not differ in relation to other cutaneous melanomas; however, due to the anatomical peculiarity of the region and the lack of specific surgical protocols, there is a tendency to perform excision with reduced safety margins, preserving the cartilage and perichondrium not affected by the tumor.^3^

It is concluded that the early diagnosis and treatment of EEMs are definitive for survival; the importance of routine clinical examination of the ears in dermatological consultations is reinforced.

## Financial support

None declared.

## Authors’ contributions

Lara martins Fiorio: Design and planning of the study; drafting and editing of the manuscript; collection, analysis, and interpretation of data; critical review of the literature.

Lucia Martins Diniz: Design and planning of the study; drafting and editing of the manuscript; effective participation in research orientation; critical review of the literature; critical review of the manuscript.

Karla Spelta: Approval of the final version of the manuscript; design and planning of the study; drafting and editing of the manuscript; collection, analysis, and interpretation of data; effective participation in research orientation; intellectual participation in propaedeutic and/or therapeutic conduct of studied cases; critical review of the literature; critical review of the manuscript.

Bruna Anjos Badaró: Design and planning of the study; drafting and editing of the manuscript; intellectual participation in propaedeutic and/or therapeutic conduct of studied cases.

## Conflicts of interest

None declared.
